# Performance of Three Prognostic Models in Patients with Cancer in Need of Intensive Care in a Medical Center in China

**DOI:** 10.1371/journal.pone.0131329

**Published:** 2015-06-25

**Authors:** XueZhong Xing, Yong Gao, HaiJun Wang, ChuLin Huang, ShiNing Qu, Hao Zhang, Hao Wang, KeLin Sun

**Affiliations:** Department of Intensive Care Unit, Cancer Hospital, Chinese Academy of Medical Sciences and Peking Union Medical College, Beijing, China; Geisel School of Medicine at Dartmouth College, UNITED STATES

## Abstract

**Objective:**

The aim of this study was to evaluate the performance of Acute Physiology and Chronic Health Evaluation II (APACHE II), Simplified Acute Physiology Score 3 (SAPS 3), and Acute Physiology and Chronic Health Evaluation IV (APACHE IV) in patients with cancer admitted to intensive care unit (ICU) in a single medical center in China.

**Materials and Methods:**

This is a retrospective observational cohort study including nine hundred and eighty one consecutive patients over a 2-year period.

**Results:**

The hospital mortality rate was 4.5%. When all 981 patients were evaluated, the area under the receiver operating characteristic curve (AUROC, 95% Confidential Intervals) of the three models in predicting hospital mortality were 0.948 (0.914–0.982), 0.863 (0.804–0.923), and 0.873 (0.813–0.934) for SAPS 3, APACHE II and APACHE IV respectively. The p values of Hosmer-Lemeshow statistics for the models were 0.759, 0.900 and 0.878 for SAPS 3, APACHE II and APACHE IV respectively. However, SAPS 3 and APACHE IV underestimated the in-hospital mortality with standardized mortality ratio (SMR) of 1.5 and 1.17 respectively, while APACHE II overestimated the in-hospital mortality with SMR of 0.72. Further analysis showed that discrimination power was better with SAPS 3 than with APACHE II and APACHE IV whether for emergency surgical and medical patients (AUROC of 0.912 vs 0.866 and 0.857) or for scheduled surgical patients (AUROC of 0.945 vs 0.834 and 0.851). Calibration was good for all models (all p > 0.05) whether for scheduled surgical patients or emergency surgical and medical patients. However, in terms of SMR, SAPS 3 was both accurate in predicting the in-hospital mortality for emergency surgical and medical patients and for scheduled surgical patients, while APACHE IV and APACHE II were not.

**Conclusion:**

In this cohort, we found that APACHE II, APACHE IV and SAPS 3 models had good discrimination and calibration ability in predicting in-hospital mortality of critically ill patients with cancer in need of intensive care. Of these three severity scores, SAPS 3 was superior to APACHE II and APACHE IV, whether in terms of discrimination and calibration power, or standardized mortality ratios.

## Introduction

The general severity-of-illness scoring systems were introduced in the field of critical care medicine in 1981[1]. Since then, numerous severity-of-illness scores have been developed for assessing critically ill patients. During the last a few years, three new general prognosis models have been developed and published: Simplified Acute Physiology Score 3 (SAPS 3) [2], Acute Physiology and Chronic Health Evaluation IV (APACHE IV) [3] and the Mortality Probability Model III (MPM III) [4]. However, studies have showed that all three models are good at presenting discrimination, but with poor calibration [5–6]. On the other hand, Acute Physiology and Chronic Health Evaluation II (APACHE II) score is still used in ICUs’ prognosis of critically ill patients [7].

Cancer patients represent 13–15% of the patients admitted to intensive care units [8–9]. Groeger et al developed the cancer mortality model in 1998[10]. However, this specific score model is not widely used since it has not proved to be superior to other models [11]. In recent years, single and multi-center studies have demonstrated that the SAPS 3 model is more accurate in the prognosis of cancer patients in need of intensive care [11–12]. However, validation of the SAPS 3 model was only conducted among critically ill cancer patients in Brazilian ICUs.

Therefore, the aim of this study is to evaluate the performances of the three prognostic models (APACHE II, APACHE IV and SAPS 3) in critically ill cancer patients in China.

## Materials and Methods

This retrospective study was conducted in the Intensive Care Unit Department of the Cancer Hospital at the Chinese Academy of Medical Sciences and Peking Union Medical College. The Cancer Hospital is the highest ranked hospital specializing in cancer in China and its ICU is a 10-bed center for the care of critically ill patients. The hospital’s Institutional Review Board approved the study and the patients’ informed consent was waived due to the observational nature of this study. Patient records/information were anonymized and de-identified prior to analysis.

The study involved patients who were admitted to the ICU between October 2008 and September 2010 but excluded those under 18 years old or with a stay at the ICU of less than 24 hours. Clinical and laboratory variables of every patient were prospectively collected by six intensivists (X. Xing, H. Wang, S. Qu, C. Huang, H. Zhang and H. Wang). Data considered for the calculation of the SAPS 3 were collected and recorded within 1 hour of ICU admission, and predicted mortality rates were calculated as recommended [2]. APACHE II and APACHE IV scores were calculated using data during the first 24 hours of admission and predicted mortality rates were calculated according to the literature [1,3]. Patients were classified based on the reason of ICU admission, i.e. medical, scheduled surgery and emergency surgery. The hospital mortality rate was the main end point.

### Ethics Statement

The Institutional Review Board (IRB) at the Cancer Hospital, Chinese Academy of Medical Sciences (ref. 11-75/510) approved this study protocol. The informed consent by patients was waived due to the observational nature of this study. The study was performed in accordance with the ethical standards laid down in the 1964 Declaration of Helsinki and its later amendments. Patient records/information were anonymized and de-identified prior to analysis.

### Statistical Analysis

Data was entered into a computer database by a single author (X. Xing). Statistical analyses were carried out using SPSS software for Windows, version 16.0 (SPSS Inc., Chicago, IL, USA). Continuous variables were presented as mean ± standard deviation or median (25–75% interquartile range) and compared, respectively, using the Student's t-test. Categorical variables were reported as absolute numbers (frequency percentages) and analyzed using χ2 test.

Statistical analysis was performed in the same way as Soares’ et al [13]. In short, validation of the prognostic scores was performed using standard tests to measure discrimination and calibration for each of the predictive models. The area under the receiver operating characteristic curve (AUROC) was used to evaluate the ability of each model to discriminate between patients who lived from those who died (discrimination). Hosmer–Lemeshow goodness-of-fit C statistic was used to evaluate the agreement between the observed and expected number of patients who did or did not die in the hospital across all of the strata of probabilities of death (calibration). A high p value (> 0.05) would indicate a good fit for the model. Calibration curves were constructed by plotting predicted mortality rates stratified by 10% intervals of mortality risk (x axis) against observed mortality rates (y axis) using Microsoft Excel software. Standardized mortality ratios (SMRs) with 95% confidential interval (CI) were calculated for each model by dividing observed by predicted mortality rates. A two-tailed p value < 0.05 was considered statistically significant.

## Results

During the study period, 1201 patients were admitted to the ICU. A total of 220 patients were excluded from the analysis due to age less than 18 years (n = 3), readmission during the same hospital (n = 49) and an ICU stay of less than 24 hours (n = 168). Therefore, 981 patients constituted the population of this study. Patients’ characteristics are displayed in Table 1. The SAPS 3 was 36.9±13.2 (range 16–102), APACHE II was 10.4±5.5 (range 0–42), and APACHE IV score was 37.7±19.8 (range 3–160) (S1 Data). Patients largely suffered from thoracic and abdominal neoplasms (80.1%), and mainly underwent scheduled surgery (92.9%).

**Table 1:** Baseline patients characteristics

Characteristics	Patients (n = 981)
**Age (years)**	64.8±12.1
**Gender (male/female)**	647/334 (53.9/46.1)
**Body mass index (Kg/m** ^**2**^ **)**	23.3±3.6
**Days prior to ICU admission (days)**	3 (1–6)
**Type of cancer (%)**	
** Thoracic neoplasms**	389 (39.7)
** Abdominal neoplasms**	396 (40.4)
** Head and neck neoplasms**	45 (4.6)
** Gynecologic and urinary neoplasms**	19 (1.9)
** Intracranial neoplasms**	29 (2.9)
** Others**	103 (10.5)
**History of radiotherapy and (or) chemotherapy**	15 (1.5)
**Cancer status (%)**	
** Solid loco-regional**	929 (94.7)
** Solid metastatic**	52 (5.3)
**Reasons for ICU admission (%)**	
** Medical**	29 (2.9)
** Schedule surgery**	911 (92.9)
** Emergency surgery**	41 (4.2)
**Mechanical ventilation (%)**	279 (28.4)
**Vasopressor (%)**	106 (10.8)
**Renal replacement therapy (%)**	13 (1.3)
**ICU length of stay (days)**	3 (2–5)
**In-hospital mortality (%)**	44 (4.5)

ICU intensive care unit

**Fig 1:**
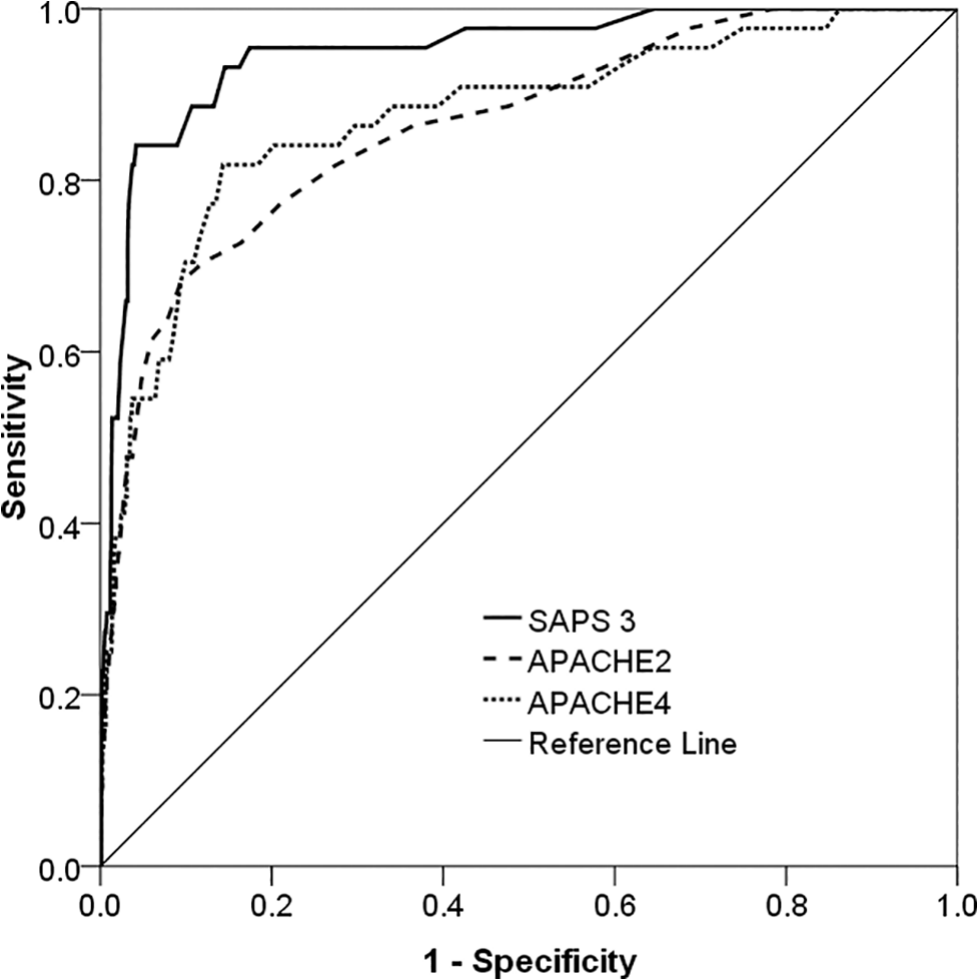
Discrimination power for three predictive models. Area under the receiver operating characteristic curve was 0.949±0.017 for SAPS 3 (p<0.001, 95% CI: 0.916–0.982), 0.863±0.030 for APACHE II (p<0.001, 95% CI: 0.804–0.923) and 0.873±0.031 for APACHE IV (p<0.001, 95% CI: 0.812–0.934). SAPS 3 Simplified Acute Physiology Score 3, APACHE II Acute Physiology and Chronic Health Evaluation II, APACHE IV Acute Physiology and Chronic Health Evaluation IV, CI confidential intervals.

**Fig 2:**
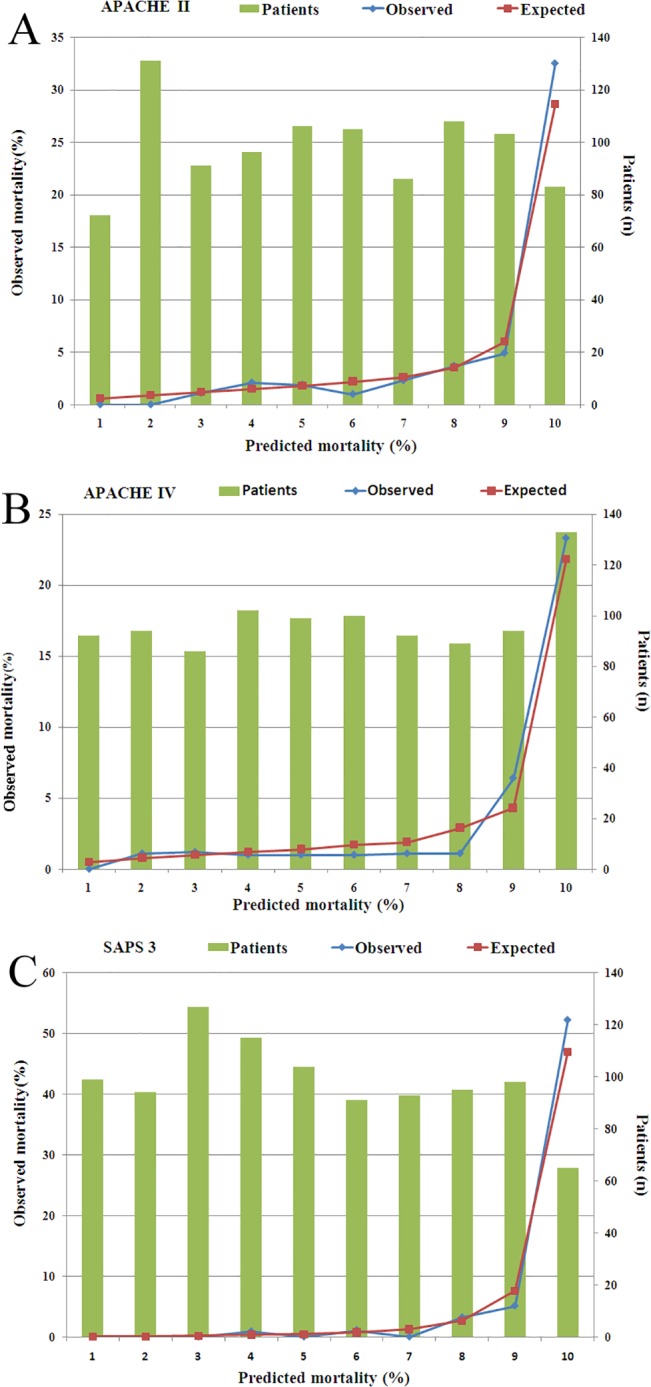
Homer-Lemeshow goodness-of fit test and calibration curves of all models. Calibration was good for all models (all p > 0.05). (A) APACHE II; (B) APACHE IV; (C) SAPS 3.

The evaluation results of the models for all 981 patients are presented in Table 2. Discrimination was very good with AUROC for all three models in all 981 patients. AUROC of SAPS 3 (0.948) was greater than those observed for APACHE II (0.863) and APACHE IV (0.873) respectively (Fig 1). Calibration was good for all models (all *P* >0.05) (Table 2 and Fig 2A–2C). Nevertheless, SAPS 3 and APACHE IV underestimated the in-hospital mortality, while APACHE II overestimated the in-hospital mortality, and with SMR <1.0 (Table 2).

**Table 2:** Performance of all models for all 981 patients.

Prognostic model	AUROC (95%CI)	p value	Goodness-of-fit test		Predicted mortality (%; median, interquartile range)	SMR
			χ^2^	p value		
All patients (n = 981; observe hospital mortality = 4.5%)						
**SAPS 3**	0.948 (0.914–0.982)	<0.001	4.987	0.759	3.00 (1.00–9.00)	1.50
**APACHE II**	0.863 (0.804–0.923)	<0.001	3.486	0.900	6.22 (4.11–10.35)	0.72
**APACHE IV**	0.873 (0.813–0.934)	<0.001	3.756	0.878	3.83 (2.08–8.70)	1.17

AUROC area under the receiver operating characteristic curve, CI confidence intervals, SMR standardized mortality ratios, SAPS 3 Simplified Acute Physiology Score 3, APACHE II Acute Physiology and Chronic Health Evaluation II, APACHE IV Acute Physiology and Chronic Health Evaluation IV

The performances of the models were further analyzed for scheduled surgical patients and emergency surgical and medical patients respectively. The results are presented in Table 3. Again, AUROC of SAPS 3 (0.912) was greater than those observed for APACHE II (0.866) and APACHE IV (0.857) in emergency surgical and medical patients, and AUROC of SAPS 3 (0.945) was greater than those observed for APACHE II (0.834) and APACHE IV (0.851) in scheduled surgical patients. This data demonstrated that discrimination power was better with SAPS 3 than with APACHE II and APACHE IV whether for emergency surgical and medical patients or for scheduled surgical patients. Calibration was good for all models (all p > 0.05) whether for scheduled surgical patients or for emergency surgical and medical patients. However, in terms of SMR, SAPS 3 and APACHE II were accurate in predicting the in-hospital mortality for emergency surgical and medical patients, while APACHE IV underestimated the in-hospital mortality. SAPS 3 and APACHE IV were accurate in predicting the in-hospital mortality for scheduled surgical patients, while APACHE II overestimated the in-hospital mortality.

**Table 3:** Performance of all models for patients receiving scheduled surgery or not

Prognostic model	AUROC (95%CI)	p value	Goodness-of-fit test		Predicted mortality (%; median, interquartile range)	SMR
			χ^2^	p value		
**Excluded schedule surgery (n = 70; observe hospital mortality = 17.1%)**						
**SAPS 3**	0.912 (0.844–0.979)	<0.001	4.890	0.769	17.00 (6.75–46.00)	1.01
**APACHE II**	0.866 (0.774–0.958)	<0.001	6.474	0.594	17.60 (9.64–32.55)	0.97
**APACHE IV**	0.857(0.762–0.952)	<0.001	6.281	0.616	10.63 (4.09–28.93)	1.61
Schedule surgery (n = 911; observe hospital mortality = 3.5%)						
**SAPS 3**	0.945 (0.902–0.987)	<0.001	4.890	0.769	3.00 (1.00–7.00)	1.17
**APACHE II**	0.834 (0.757–0.911)	<0.001	6.474	0.594	6.05 (4.10–9.47)	0.58
**APACHE IV**	0.851(0.772–0.930)	<0.001	6.281	0.616	3.62 (2.02–7.83)	0.97

AUROC area under the receiver operating characteristic curve, CI confidence intervals, SMR standardized mortality ratios, SAPS 3 Simplified Acute Physiology Score 3, APACHE II Acute Physiology and Chronic Health Evaluation II, APACHE IV Acute Physiology and Chronic Health Evaluation IV

## Discussion

During the study, we found that APACHE II, APACHE IV and SAPS 3 models had excellent discrimination and calibration power. In terms of SMR, SAPS 3 was more accurate in predicting the in-hospital mortality than APACHE II and APACHE IV, whether for emergency surgical and medical patients or for scheduled surgical patients.

To our knowledge, this is the first study exploring the validation of APACHE II, APACHE IV and SAPS 3 models in cancer patients in China, regardless of whether they have had surgery or not. We chose APACHE II for comparison with APACHE IV and SAPS 3 models because APACHE II is currently used in our ICU and is the most popular model in China [9,14]. Many China ICUs are reluctant to implement APACHE IV and SAPS 3 models due to the greater familiarity with APACHE II and the lack of validation studies with APACHE IV and SAPS 3 in China. With the introduction of the SAPS 3 model in 2005[2] and the APACHE IV model in 2006[3], it has been suggested that the older models should no longer be used because they become increasingly inaccurate [3]. Good discrimination and calibration of SAPS 3, in particular customized equation of SAPS 3, have been reported in critically ill cancer patients in single and multicenter studies in Brazilian ICUs [11–12]. Finally, we choose APACHE IV because the performance of the APACHE IV model offered excellent discrimination and calibration in a large common dataset [15], but it has not yet been validated in cancer patients in China. We did not choose the CMM model because it overestimates mortality rates regardless of studying elective surgical patients or not, and previous studies have not shown improvement of the mortality prediction in comparison with general scores [11].

The APACHE II model is still widely used all over the world whether in general or academic ICUs [16–21]. In this study, we found that AUROC of APACHE II is 0.863 in all 981 patients, which is in accordance with those reported by most authors [16,18–20]. However, it overestimated the in-hospital mortality rate with SMR of 0.72, although its calibration was good, with a p value of 0.900. After excluding scheduled surgical patients, it was accurate in predicting the hospital mortality for emergency surgical and medical patients. Surgical patients had temporary physiological derangement due to the effects of anesthesia. Therefore, it was not surprising that the use of APACHE II scores led to an overestimation of mortality rates in surgical patients [22]. Customization or adding new variables may improve the calibration power. Chang et al incorporated metastasis and respiratory failure variables into the APACHE II model and found that the AUROC of APACHE II score for medical patients increased from 0.82 to 0.86, and the fit of the modified model was excellent compared with the APACHE II model alone [22].

The APACHE IV model was developed using a very large database in the United States [3] and several validation studies have been reported [6,15–16,19]. Not surprisingly, the APACHE IV model offered the best discrimination and calibration mainly in U.S. ICU patients[15], but poor calibration for patients outside the U.S., although it showed good discrimination[16,19]. In our study, we found that AUROC of APACHE IV is 0.873 in all 981 patients, which is in accordance with those reported by most authors [6,15–16,19]. However, it underestimated the in-hospital mortality rate with SMR of 1.17, although calibration of it was good with a p value of 0.878. Its calibration power declined from 1.17 to 1.61 for emergency surgical and medical patients. However, this was not the case for scheduled surgical patients, and the calibration ability of APACHE IV model for the latter was 0.97 in terms of SMR, which demonstrated good calibration. Overall, the new APACHE IV scoring system performed better than older counterparts of APACHE II due to the introduction of more predictive variables [6]. In developing countries, however, the burden of manual data collection of a lot of variables may become relevant due to a shortage of electronic charting, which may partly hinder the choice and use of new scoring models. As a result, how to balance the complex and ease of use of new scoring systems is a challenge.

Single and multicenter validation studies led by Soares et al demonstrated that the SAPS 3 prognostic model was accurate in predicting outcomes in critically ill patients with cancer in need of intensive care [11–12]. In their studies, both discrimination and calibration were good for non-scheduled surgical patients for CSA (customized equation for countries from Central and South America) SAPS 3 but not for SAPS 3. In our study, we chose SAPS 3 for validation as general SAPS 3 exhibited good calibration and modest discrimination in Asian critically ill patients [23]. In this study, we found that SAPS 3 had better discrimination ability than APACHE II and APACHE IV models, and all models had good calibration power. In terms of SMR, SAPS 3 was more accurate in predicting the in-hospital mortality than APACHE II and APACHE IV whether for emergency surgical and medical patients or for scheduled surgical patients.

Most studies reported similarly good discriminative capabilities of all prognostic models but conflicting results regarding calibration [5–6,15–16,19]. Peek et al found that calibration tests were extremely sensitive to sample size [24]. In their study, they found that in the calibration tests, the frequency of agreement rose from 78% (250 observations) to 86% (750 observations) and 93% (1000 observations). However, after customization, the Hosmer-Lemeshow test accepted the model in the majority of cases (99% with a sample size of 250, 89% with a sample size of 5000). Therefore, it may be appropriate that local customization is mandatory to improve the calibration ability of prognostic models.

Severity of illness scoring systems have been designed for benchmarking, performance improvement, resource use, and clinical decision support [25]. One recent study showed that forty percent of 40933 patients had a mortality risk of less than 10% and did not have an intensive treatment such as mechanical ventilation, noninvasive ventilation, blood product administration, renal replacement therapy, or treatment with a vasoactive medication [26]. As ICU is a place for the most critically ill patients, research has been done to study better triage decisions other than severity of illness. A preliminary study showed that the application of advances in health information technology (HIT) might contribute to better triage decisions [27]. Until now, current outcome prediction models have increasingly focused on benchmarks for resource use [28].

Our study has potential limitations. Firstly, this was a single center study and only critically ill cancer patients were included. Therefore, the result of this study may not be generalized to other general medical centers. Secondly, local customization may provide a better calibration, therefore further investigations should be undertaken to evaluate second level customization of all prognostic models in critically ill patients with cancer. Thirdly, there were no patients who had leukemia or lymphoma, and few patients suffered metastatic solid tumors. Therefore, the result of this study was similar to that of general surgical ICU studies [29]. Finally, the overall hospital mortality rate was very low although our cohort included 981 patients. This might have an impact on the performance of all prognostic models.

## Conclusions

In this cohort, we found that APACHE II, APACHE IV and SAPS 3 models had good discrimination and calibration ability in predicting in-hospital mortality of critically ill patients with cancer in need of intensive care. Of these three severity scores, SAPS 3 was superior to APACHE II and APACHE IV, whether in terms of discrimination and calibration power, or standardized mortality ratios.

## Supporting Information

S1 Data Part of raw data of all 981 patients. (RAR)
